# Effects of Rho inhibitors on membrane depolarization‐induced contraction of male rat caudal arterial smooth muscle

**DOI:** 10.14814/phy2.70293

**Published:** 2025-04-01

**Authors:** Kazuki Aida, Reiko Ishii‐Nozawa, Mitsuo Mita

**Affiliations:** ^1^ Department of Pharmacology Meiji Pharmaceutical University Tokyo Japan; ^2^ Department of Cardiovascular Pharmacology, Education and Research Unit for Comprehensive Clinical Pharmacy Meiji Pharmaceutical University Tokyo Japan

**Keywords:** proline‐rich tyrosine kinase 2, Rho guanine nucleotide exchange factor, RhoA, Rhosin, Y16

## Abstract

We previously reported that depolarization of the vascular smooth muscle plasma membrane activates the Ca^2+^‐dependent proline‐rich tyrosine kinase 2 (Pyk2) upstream of the RhoA/Rho‐associated kinase (ROCK) pathway, leading to phosphorylation of the myosin‐targeting subunit of myosin light chain phosphatase (MYPT1) and the 20 kDa light chain of myosin (LC_20_). However, the mechanism whereby Pyk2 activates RhoA remains unclear. It is conceivable that Rho guanine nucleotide exchange factors (RhoGEFs) may link activated Pyk2 to RhoA activation through phosphorylation and activation of RhoGEFs. In this study, we investigated the activation of RhoA and RhoGEFs in membrane depolarization‐induced contraction of rat caudal arterial smooth muscle. Rhosin, a RhoA inhibitor, concentration‐dependently inhibited both the phasic and tonic components of the 60 mM K^+^‐induced contraction, and the inhibition was particularly prominent in the tonic contraction. On the contrary, Y16, a RhoGEF inhibitor, had little inhibitory effect. Moreover, phosphorylation of MYPT1 was increased at Thr697 and Thr855 by 60 mM K^+^ stimulation for 15 min, and this increase in MYPT1 phosphorylation was inhibited in the presence of Rhosin, but not Y16. We conclude that Pyk2 activated in response to Ca^2+^ entry induced by depolarization may cause activation of Y16‐insensitive RhoGEFs and RhoA, resulting in sustained contraction.

## 1 INTRODUCTION

Vascular smooth muscle contraction is controlled by the balance between phosphorylation and dephosphorylation of the 20 kDa light chains of myosin (LC_20_) (Hartshorne et al., 1998; Kamm & Stull, 2001; Somlyo & Somlyo, 2003). Phosphorylation and dephosphorylation of LC_20_ are catalyzed by Ca^2+^/calmodulin (CaM)‐dependent myosin light chain kinase (MLCK) and myosin light chain phosphatase (MLCP), respectively (Hartshorne et al., 1998; Kamm & Stull, 2001; Somlyo & Somlyo, 2003). In contrast, the molecular mechanisms that regulate force independent of changes in cytoplasmic free Ca^2+^ concentration ([Ca^2+^]_i_), so‐called Ca^2+^ sensitization, has recently attracted attention (Somlyo & Somlyo, 2003). Ca^2+^ sensitization occurs mainly through inhibition of MLCP, leading to an increase in phosphorylation of LC_20_ (Somlyo & Somlyo, 2003). RhoA plays a crucial role in Ca^2+^ sensitization: RhoA‐GTP activates Rho‐associated kinase (ROCK), resulting in phosphorylation of the myosin‐targeting subunit of MLCP (MYPT1) (Somlyo & Somlyo, 2003). This inactivates the phosphatase, thereby promoting the phosphorylation of LC_20_ and enhancing contraction (Hartshorne et al., 1998; Somlyo & Somlyo, 2003). Therefore, the activation of the RhoA/ROCK pathway serves as a major downstream signaling pathway for receptor and G protein‐mediated Ca^2+^ sensitization (Somlyo & Somlyo, 2003; Swärd et al., 2003).

Electromechanical coupling works through changes in membrane potential, affecting [Ca^2+^]_i_. Stimulation by high [K^+^] induces depolarization of the cell membrane, opening voltage‐gated Ca^2+^ channels and causing Ca^2+^ influx. Increased [Ca^2+^]_i_ binds to CaM, leading to activation of MLCK, phosphorylation of LC_20_, and contraction (Bolton et al., 1999; Somlyo & Somlyo, 2003). We have previously shown that membrane depolarization induced by high [K^+^] stimulation rapidly increased the force of denuded rat tail arterial smooth muscle, followed by a decline to a steady‐state level that was greater than resting force (Mita et al., 2002). The phasic contraction is due to an increasing [Ca^2+^]_i_ and the LC_20_ phosphorylation catalyzed by MLCK, whereas the sustained contraction accompanies with the activation of the RhoA/ROCK pathway, leading to MLCP inhibition (Mita et al., 2002). In addition, we recently demonstrated that the Ca^2+^‐dependent proline‐rich tyrosine kinase 2 (Pyk2) is upstream of RhoA activation by high [K^+^]‐induced membrane depolarization and a Ca^2+^ ionophore, ionomycin in rat caudal arterial smooth muscle, leading to MLCP inhibition and sustained contraction (Mita et al., 2013; Mills, Mita, Nakagawa, et al., 2015; Mills, Mita & Walsh, 2015; Aida et al., 2024).

Small GTPase Rho proteins act as biomolecular switches by adopting different conformational states upon binding of GDP or GTP. Rho activity is facilitated by guanine nucleotide exchange factors (RhoGEFs), which catalyze the exchange of GDP for GTP in response to various extracellular stimuli (Jaffe & Hall, 2005) and finally regulate many cellular responses such as cell morphology, growth, and motility (Rossman et al., 2005). In cardiovascular disorders, including hypertension, RhoGEFs could be considered as key molecules involved in the hyperactivity of RhoA (Cario‐Toumaniantz et al., 2012; Loirand et al., 2008; Strassheim et al., 2019). Nevertheless, knowledge of the expression, activity, and regulation of RhoGEFs in vascular smooth muscle cells is still limited. It has been reported that the three members of the regulators of G protein signaling (RGS)‐containing RhoGEF subfamily of RhoGEFs, namely Arhgef1 (p115‐RhoGEF), Arhgef11 (PDZ‐RhoGEF), and Arhgef12 (Leukemia‐associated RhoGEF; LARG), are expressed in arteries, notably with a higher expression of PDZ‐RhoGEF at both mRNA and protein levels in rat aorta and mesenteric artery (Cario‐Toumaniantz et al., 2012; Jin et al., 2006; Ying et al., 2006). In contrast, in mouse aorta, LARG has been reported to be the predominant RGS‐RhoGEF, and PDZ‐RhoGEF was less expressed (Wirth et al., 2008). The types of RhoGEF expressed differ depending on the tissue, but details have not been clarified.

The mechanism by which Pyk2 involved in membrane depolarization‐induced contraction activates the RhoA/ROCK pathways remains unknown. Therefore, in this study, we used two Rho inhibitors, Rhosin and Y16, with different sites of action, to clarify the involvement of RhoGEFs in the Pyk2‐mediated Ca^2+^‐dependent RhoA/ROCK pathway in membrane depolarization‐induced‐sustained contraction of rat caudal arterial smooth muscle.

## 2 MATERIALS AND METHODS

### 2.1 Materials

Prazosin (1‐[4‐amino‐6,7‐dimethoxy‐2‐quinazolinyl]‐4‐[2‐furanylcarbonyl]‐ piperazine) (Catalog #P7791), DL‐propranolol (1‐[isopropylamino]‐3‐[1‐naphthyloxy]‐2‐propanol) (Catalog #P0884), Rhosin ((2*R*)‐2‐Amino‐3‐(1H‐indol‐3‐yl)‐*N*′‐((1*E*)‐quinoxalin‐6‐ylmethylidene)) propanehydrazide (Catalog #555460) and Y16 (4‐[[3‐[(3‐Methylphenyl) methoxy]phenyl]methylene]‐1‐phenyl‐3,5‐pyrazolidinedione) (Catalog #504043) were purchased from Sigma‐Aldrich (St. Louis, MO, USA), dithiothreitol (DTT) (Catalog #045–08974) from Wako Pure Chemical Industries (Wako) (Osaka, Japan) and 2‐[4‐(2‐Hydroxyethyl)‐1‐piperazinyl]ethanesulfonic acid (Hepes) (Catalog #342–01375) from Dojindo Laboratories (Kumamoto, Japan). All other chemicals were of reagent grade. Stock solutions were prepared in water for prazosin and propranolol, and in dimethyl sulfoxide (DMSO) (Wako, Catalog #043–07216) for Rhosin and Y16.

### 2.2 Force measurements in intact muscle strips

This study conforms to Guidelines for Proper Conduct of Animal Experiments in Japan. Male Sprague–Dawley rats (500–550 g) were anesthetized with 5% isoflurane and euthanized by carbon dioxide as approved by the Institutional Ethics Committee for Animal Research at Meiji Pharmaceutical University. De‐endothelialized caudal arterial helical strips were prepared for force measurements as previously described (Aida et al., 2024; Mita & Walsh, 1997). Muscle strips were mounted horizontally between two hooks and immersed in a pool of Hepes‐Tyrode (H‐T solution) (137 mM NaCl (Wako, Catalog #191–01665), 2.7 mM KCl (Wako, Catalog #163–03545), 1.8 mM CaCl_2_ (Wako, Catalog #038–24985), 1 mM MgCl_2_ (Wako, Catalog #135–00165), 5.6 mM glucose (Wako, Catalog #049–31165), 10 mM Hepes, pH 7.4) with a transducer attached to one of the hook. Once mounted, tissues were stimulated with 60 mM KCl by replacing NaCl in H‐T solution with equimolar KCl, which was adjusted to pH 7.4. The absence of vasorelaxation to 1 μM acetylcholine (Daiichi‐Sankyo, OVISOT® FOR INJECTION, JAN code #4987081104116) indicated successful endothelial cell disruption. All buffers were pre‐oxygenated with 100% O_2_ at room temperature. All measurements of 60 mM K^+^‐induced contraction were carried out in the presence of 1 μM prazosin and 0.1 μM propranolol to block the α_1_‐ and β‐adrenergic effects of noradrenaline, which is released from nerve terminals by depolarization (Mita et al., 2002). Control contractions were obtained after stable and identical 60 mM K^+^‐induced contractions were achieved in the presence of 1 μM prazosin and 0.1 μM propranolol. Subsequently, the strips were transferred to H‐T solution in the presence of 1 μM prazosin and 0.1 μM propranolol for 45 min before transfer to the test solutions with Rhosin or Y16 (containing 1 μM prazosin and 0.1 μM propranolol). Each inhibitor was applied for 30 min before the transfer to 60 mM K^+^. Rhosin and Y16 were dissolved in DMSO as a vehicle in all experiments, with the maximum concentration of DMSO being 0.35%. We previously reported that treatment with DMSO alone (up to 1%) had no effect on 60 mM K^+^‐induced contraction (Mita et al., 2002). Therefore, in the experiment examining the effect of inhibitors on 60 mM K^+^‐induced contraction, contractions in the presence of each inhibitor were compared with the control contractions described above as the control group. In all experiments, data were obtained from tail arterial strips isolated from several rats rather than from only one rat.

### 2.3 Western blotting of MYPT1

Tissues were treated with vehicle (DMSO) or each inhibitor for 30 min. Then, before or 15 min after 60 mM K^+^ stimulation, tissues were rapidly frozen in 10% (w/v) trichloroacetic acid (TCA) (Wako, Catalog #204–02405) and 10 mM DTT in dry ice/acetone (Wako, Catalog #012–00343). Tissues were lyophilized for 16 h and stored at −80°C until extraction of proteins. Extraction of proteins was achieved by the methods of Wilson et al. (Wilson et al., 2005) and Mita et al. (Mita et al., 2013). Protein was extracted from freeze‐dried tissues by the addition of 72 μL of 50 mM Tris–HCl (Sigma‐Aldrich, Catalog #T1503), pH 6.8, containing 1% SDS (Bio‐Rad, Catalog #1610301) and a 1% protease inhibitor cocktail (Sigma‐Aldrich, Catalog #P8340). Samples were heated to 95°C for 5 min, and then mixed for 60 min. Samples (15 μL) were subjected to SDS‐PAGE (7.5% acrylamide). After transfer to nitrocellulose membrane (Bio‐Rad, Catalog #162–0150), the membranes were blocked with 1% Blocking Reagent (Roche, Mannheim, Germany, Catalog #11500694001) in TBS (50 mM Tris, 150 mM NaCl, pH 7.5) for 2 h at room temperature and then incubated with primary antibody for 16 h at 4°C and secondary antibody for 2 h at room temperature. Blotted proteins were detected with the enhanced chemiluminescence detection and quantitated by densitometry. The level of phosphorylation of MYPT1 at Thr697 and Thr855 was calculated according to P‐Thr697 MYPT1 or P‐Thr855 MYPT1/total MYPT1. Anti‐MYPT1 rabbit polyclonal antibody (Merck‐Millipore, Billerica, MA, USA, Catalog #07–672) was used at 1:2000 dilution. Anti‐[phosphoThr697]‐MYPT1 rabbit polyclonal antibody (Merck‐Millipore, Catalog #07–251) was used at 1:7000 dilution. Anti‐[phosphoThr855]‐MYPT1 rabbit polyclonal antibody (Upstate Biotechnology, Charlottesville, VA, USA, Catalog #36–003) was used at 1:2000 dilution. Anti‐mouse/rabbit IgG‐POD antibody (Roche, Catalog #11520709001) was used at 1:10000 dilution (for Total MYPT1), or 1:7500 dilution (for [phosphoThr697] and [phosphoThr855] MYPT1).

### 2.4 Statistical analysis

Data represent the mean ± standard error of the mean (SEM), except for continuous data which represent the mean ± standard deviation (SD). The values of *N* indicate the number of rats, and the values of *n* indicate the number of smooth muscle strips utilized. Student's *t*‐test was used for statistical comparisons. One‐way ANOVA followed by Dunnett's multiple comparison post hoc test was used to compare three or more groups. *p*‐value <0.05 was considered significant. These statistical analyses were performed with a computer program (JMP Pro16, SAS Institute Japan, Tokyo, Japan).

## 3 RESULTS

We tested the effects of Rhosin, a RhoA inhibitor, and Y16, a RhoGEF inhibitor, on 60 mM K^+^‐induced contraction of rat caudal arterial smooth muscle in the same strips. Rhosin and Y16 were not soluble in H‐T solution above 30 μM, so concentrations up to 30 μM were used in all experiments.

**FIGURE 1:**
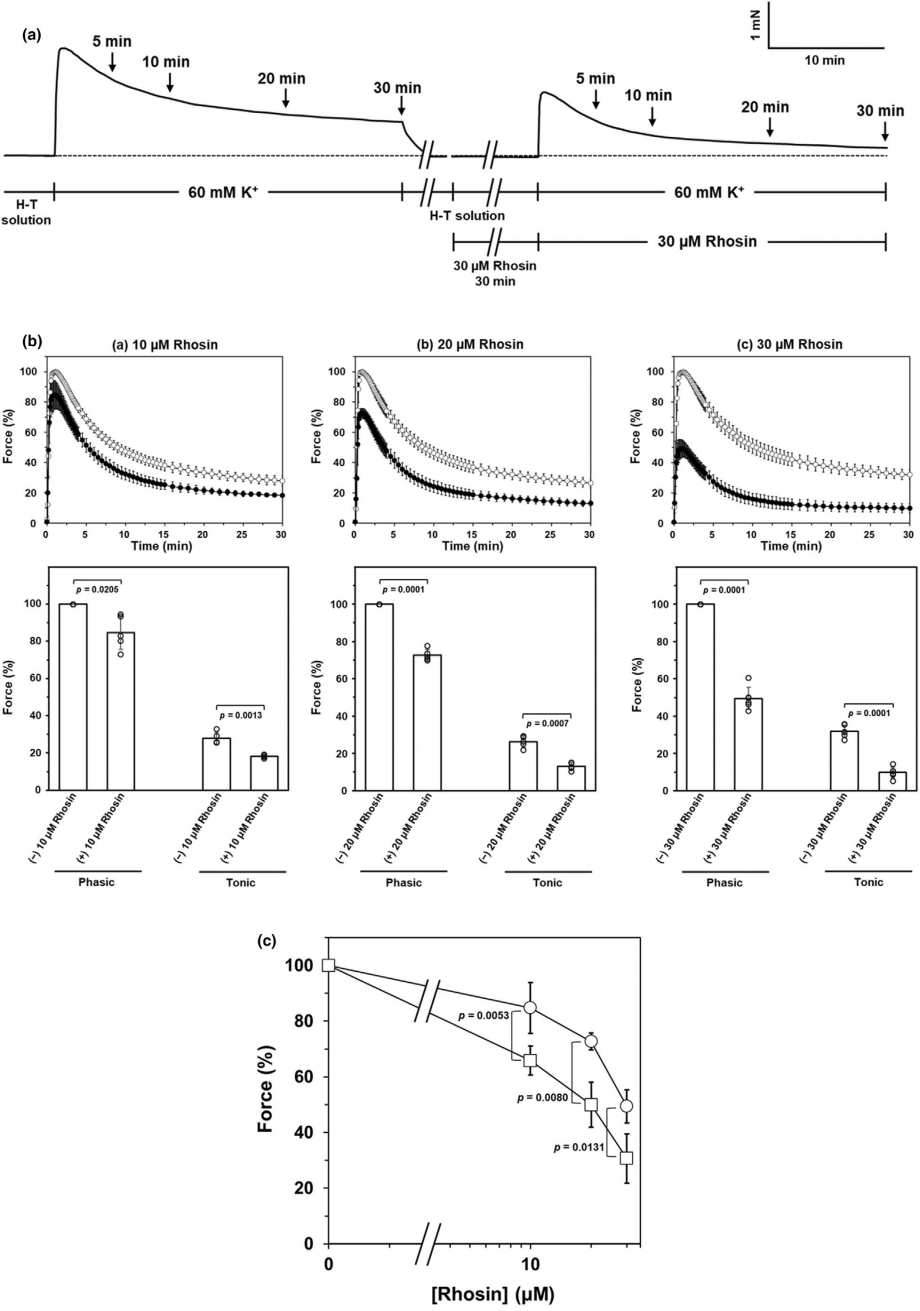
Effect of Rhosin on K^+^‐induced contraction of rat caudal arterial smooth muscle (a) A typical trace showing the effect of pre‐treatment with 30 μM Rhosin on 60 mM K^+^‐induced contraction of rat caudal arterial smooth muscle. The trace is representative of six experiments. (b) Upper panels show the time courses of 60 mM K^+^‐induced contraction without (○) or with (●) Rhosin pre‐treatment ((a)—(c) 10 μM, 20 μM, and 30 μM Rhosin pre‐treatment, respectively). Lower panels show the effect of Rhosin on the phasic and tonic components of 60 mM K^+^‐induced contraction. Force is expressed as a percentage of the maximal force of the phasic contraction induced by 60 mM K^+^ without Rhosin. Values represent the mean ± SD. (*N* = 2, *n* = 5 for 10 μM and 20 μM Rhosin; *N* = 2, *n* = 6 for 30 μM Rhosin). Significance was tested by the Student's paired *t*‐test. (C) Concentration‐dependent inhibitory effect of Rhosin on the phasic and tonic components of K^+^‐induced contraction. Force is expressed as a percentage of the maximal force in the phasic component (○) of the 60 mM K^+^‐induced contraction, or as a percentage of the force 30 min after 60 mM K^+^ stimulation in the tonic component (□), respectively, in the absence of Rhosin. Significance was tested by the Student's unpaired *t*‐test.

**FIGURE 2:**
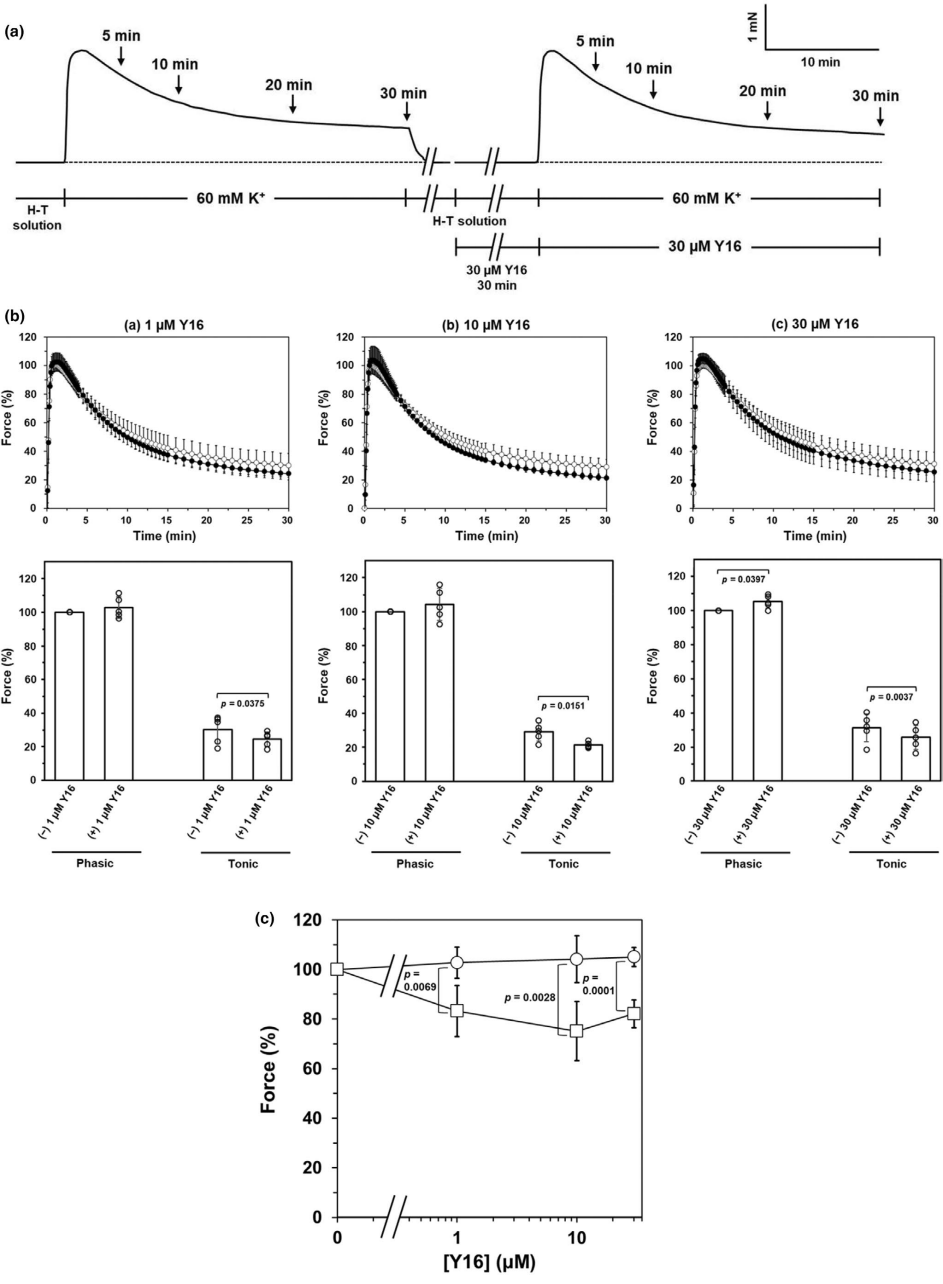
Effect of Y16 on K^+^‐induced contraction of rat caudal arterial smooth muscle (a) A typical trace showing the effect of pre‐treatment with 30 μM Y16 on 60 mM K^+^‐induced contraction of rat caudal arterial smooth muscle. The trace is representative of six experiments. (b) Upper panels show the time courses of 60 mM K^+^‐induced contraction without (○) or with (●) Y16 pre‐treatment ((a)—(c) 1 μM, 10 μM and 30 μM Y16 pre‐treatment, respectively). Lower panels show the effects of Y16 on the phasic and tonic components of 60 mM K^+^‐induced contraction. Force is expressed as a percentage of the maximal force of the phasic contraction induced by 60 mM K^+^ without Y16. Values represent the mean ± SD. (*N* = 2, *n* = 5). Significance was tested by the Student's paired *t*‐test. (c) Concentration‐dependent inhibitory effect of Y16 on the phasic and tonic components of K^+^‐induced contraction. Force is expressed as a percentage of the maximal force in the phasic component (○) of the 60 mM K^+^‐induced contraction, or as a percentage of the force 30 min after 60 mM K^+^ stimulation in the tonic component (□), respectively, in the absence of Y16. Significance was tested by the Student's unpaired *t*‐test.

The average maximal force of the phasic contraction developed in response to 60 mM K^+^ in the absence of inhibitors was 1.88 ± 0.46 mN/mg tissue wet weight (*n* = 41). Pre‐treatment with Rhosin suppressed 60 mM K^+^‐induced phasic and tonic contraction in a concentration‐dependent manner (Figure 1a,b). However, the inhibitory effect of Rhosin on tonic contractions is greater than that on phasic contractions (Figure 1c). In contrast, pre‐treatment with Y16 had no effect on 60 mM K^+^‐induced contraction, except for slight inhibition of the tonic contraction induced by 60 mM K^+^ (Figure 2a,b). Y16 significantly inhibited the tonic contraction compared with the contraction in the absence of Y16, but the inhibition was slight and not concentration‐dependent (Figure 2c), and even at the highest concentration of 30 μM Y16, the tonic component of 60 mM K^+^‐induced contraction was 82.2 ± 5.6% of the tonic contraction in the absence of Y16 (Figure 2c).

**FIGURE 3:**
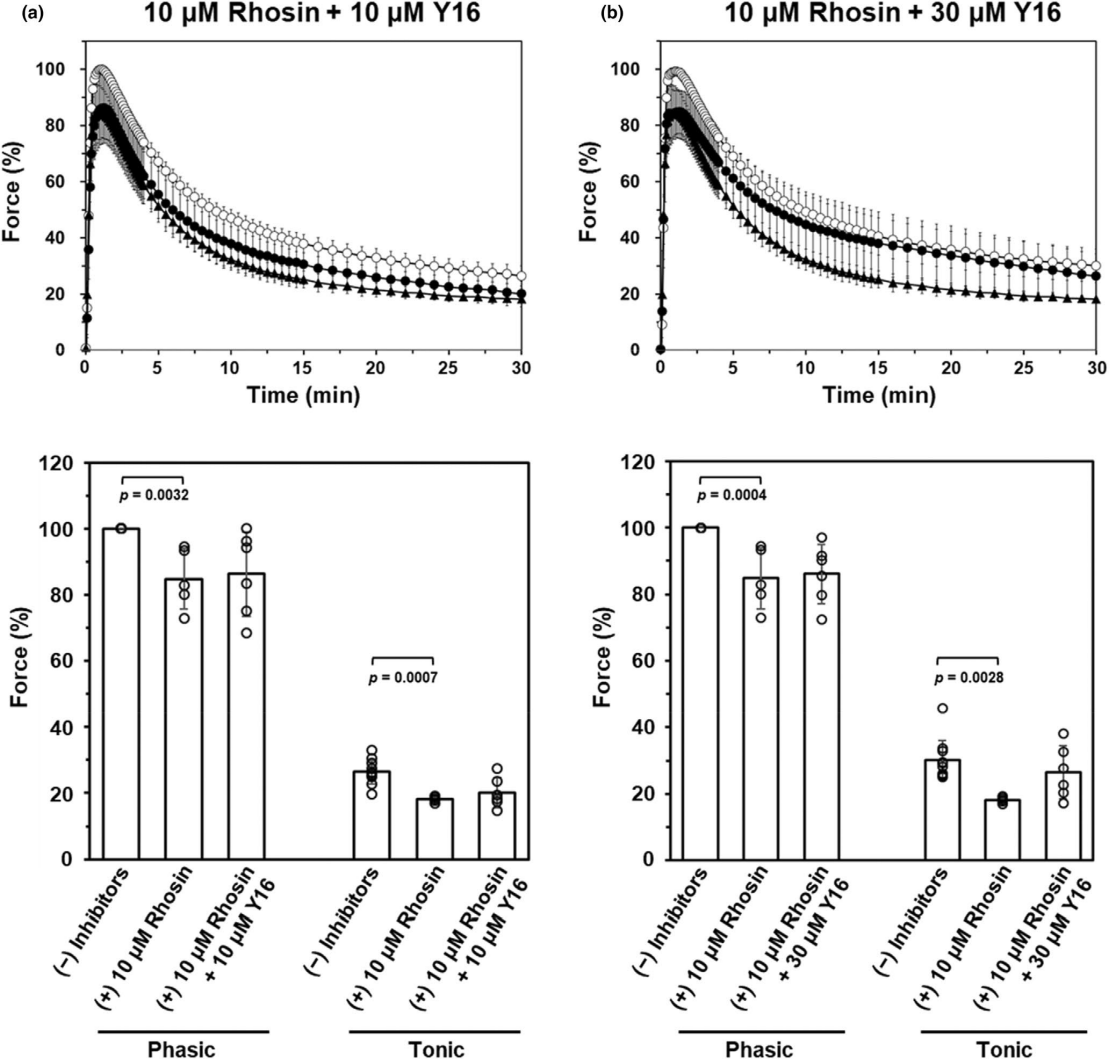
Effects of Rhosin in combination with Y16 on K^+^‐induced contractions of rat caudal arterial smooth muscle Rat caudal strips were pre‐treated with Rhosin + Y16 at the indicated concentrations for 30 min. Upper panels show the time courses of 60 mM K^+^‐induced contraction without inhibitors (○), with 10 μM Rhosin alone (▲) or with 10 μM Rhosin + Y16 pre‐treatment (●) ((a) 10 μM Rhosin + 10 μM Y16; (b) 10 μM Rhosin + 30 μM Y16). Lower panels show the effects of Rhosin + Y16 on the phasic, and tonic components of 60 mM K^+^‐induced contraction. Force is expressed as a percentage of the maximal force of the phasic contraction induced by 60 mM K^+^ without Rhosin or Rhosin + Y16. Values represent the mean ± SD. (*N* = 8, *n* = 11 for no inhibitors, *N* = 4, *n* = 5 for 10 μM Rhosin alone; *N* = 4, *n* = 6 for 10 μM Rhosin + 10 μM Y16 or 10 μM Rhosin + 30 μM Y16). Statistical comparisons of means between groups were performed using one‐way ANOVA followed by Dunnett's multiple comparison post hoc test compared with 10 μM Rhosin alone.

The inhibitory effects of combined Rhosin and Y16 were also examined. Pre‐treatment with 10 μM Rhosin slightly, but significantly, inhibited 60 mM K^+^‐induced tonic and phasic contraction, as shown in Figure 1. Therefore, we decided that 10 μM Rhosin was suitable to easily confirm the synergistic effect of Y16. When 10 μM Rhosin was combined with 10 μM or 30 μM Y16, the inhibitory effect on K^+^‐induced contraction was not affected, compared with 10 μM Rhosin alone (Figure 3).

**FIGURE 4:**
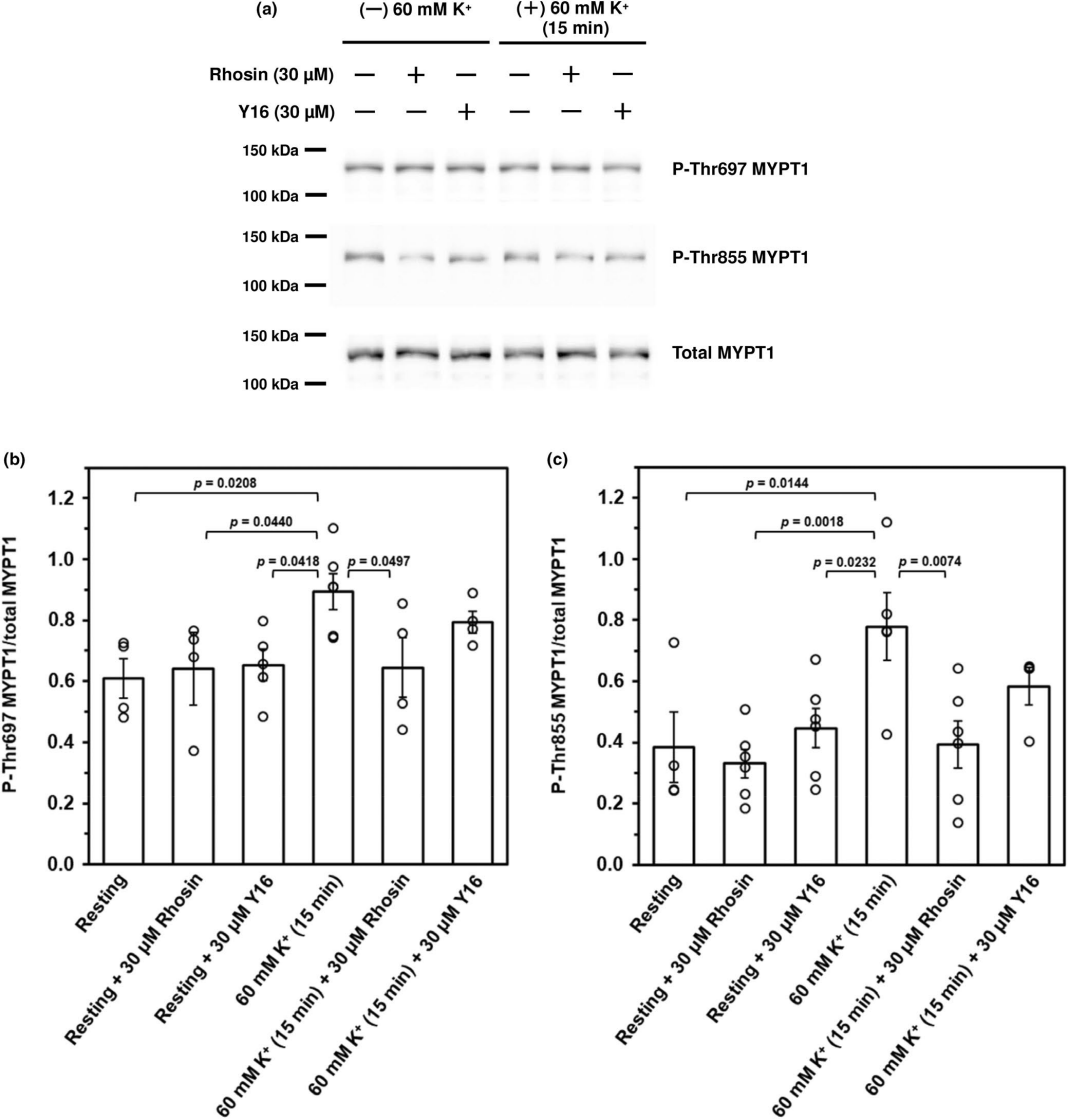
Effects of Rhosin and Y16 on K^+^‐induced phosphorylation of MYPT1 at Thr697 and Thr855 (a) Representative western blots showing 60 mM K^+^‐induced phosphorylation of MYPT1 at Thr697 and Thr855 in the absence or presence of 30 μM Rhosin or Y16. (b, c) Cumulative data of the phosphorylation of MYPT1 at Thr697 (b) and Thr855 (c) in the absence or presence of 30 μM Rhosin or Y16. Values represent the mean ± SEM (*N* = 6, *n* = 4–6). Statistical comparisons of means between groups were performed using one‐way ANOVA followed by Dunnett's multiple comparison post hoc test compared with 60 mM K^+^ stimulation group in the absence of inhibitors.

Phosphorylation of MYPT1 at Thr697 and Thr855 in caudal arterial smooth muscle caused by 60 mM K^+^ stimulation was investigated using phosphospecific antibodies. We previously reported that treatment with 60 mM K^+^ for 15 min significantly increased phosphorylation at both Thr697 and Thr855 (Mills, Mita, Nakagawa, et al., 2015; Mita et al., 2013). Therefore, we investigated the phosphorylation of MYPT1 at Thr697 and Thr855 induced by 60 mM K^+^ stimulation for 15 min, following treatment with the maximal concentration of Rhosin, Y16, or vehicle (DMSO) used in contraction experiments for 30 min. Basal phosphorylation was detected at Thr697 and Thr855 in unstimulated tissue, and treatment with 60 mM K^+^ for 15 min significantly increased phosphorylation at both Thr697 and Thr855 compared with basal phosphorylation at Thr697 and Thr855 (Figure 4). The increase in MYPT1 phosphorylation at Thr697 and Thr855 induced by 60 mM K^+^ stimulation for 15 min was completely abolished in the presence of 30 μM Rhosin (Figure 4). On the contrary, 30 μM Y16 tended to slightly inhibit the 60 mM K^+^‐caused increase in MYPT1 phosphorylation at Thr697 and Thr855; however, the inhibition was not significant compared with the 60 mM K^+^‐induced increase in MYPT1 phosphorylation in the absence of inhibitors (Figure 4).

## 4 DISCUSSION

In this study, we aimed to elucidate the involvement of RhoGEF in the RhoA/ROCK activation mediated by Pyk2 in response to high [K^+^] stimulation, using two RhoA inhibitors with different mechanisms of action, Rhosin and Y16.

RhoA activation is considered to be affected by RhoGEFs and turned off by Rho GTPase‐activating proteins (RhoGAPs) (Rossman et al., 2005). However, the involvement of RhoGEFs or RhoGAPs in Pyk2‐mediated RhoA/ROCK pathway activation has not been elucidated. One possibility arises that RhoGEFs or RhoGAPs, which are known to be tyrosine phosphorylated (Burridge & Chrzanowska‐Wodnicka, 1996; Kato et al., 2000; Roof et al., 1998), may be altered by activated Pyk2 in response to the sustained increase in [Ca^2+^]_i_ upon membrane depolarization, leading to activation of RhoGEFs or inhibition of RhoGAPs. Therefore, in this study, we examined the effects of Rhosin and Y16 on the contraction and MYPT1 phosphorylation induced by high [K^+^] in de‐endothelialized helical strips of the rat caudal artery. Although several reports have been published on the effects of Rhosin and Y16 on cultured cells, including smooth muscle cells, our research is the first study to investigate the effects of Rhosin and Y16 on intact smooth muscle tissue.

Y16 is a cell‐permeable inhibitor of RhoGEFs that is shown to target the RhoGEF DH‐PH domain junction with high affinity (*K*_d_ = 65 nM) and effectively prevent RhoGEFs (LARG, p115‐RhoGEF, and PDZ‐RhoGEF) from interacting with RhoA (Shang et al., 2012; Shang et al., 2013). 10 μM Y16 was indicated to completely prevent serum‐induced activation of cellular RhoA, but not Cdc42 or Rac1, and RhoA downstream signaling events in NIH‐3 T3 cell cultures (Shang et al., 2013). Specifically, Y16 binds to the RhoA‐binding site of RhoGEF and inhibits RhoA activation catalyzed by RhoGEF (Shang et al., 2013). In contrast, Rhosin specifically binds to the RhoGEF‐binding domain of RhoA (*K*_d_ = 354 nM) and inhibits GEF‐catalyzed RhoA activation (Shang et al., 2012). Moreover, Rhosin concentration‐dependently reduced RhoA and myosin phosphorylation activities of MCF7 cell‐derived mammospheres with an EC_50_ value of 30–50 μM, and decreased the size and number of mammospheres in MCF7 cells (Shang et al., 2012). Therefore, it has been reported that Y16 acts synergistically with Rhosin to inhibit LARG–RhoA interaction, RhoA activation, and RhoA‐mediated signaling functions (Shang et al., 2013).

Rhosin inhibited concentration‐dependently 60 mM K^+^‐induced contraction of rat caudal arterial smooth muscle strips, particularly 60 mM K^+^‐induced‐sustained contraction (Figure 1). On the contrary, Y16 did not inhibit K^+^‐induced contraction, except for a small inhibition of the tonic component of contraction without concentration dependence (Figure 2). Activation of the RhoA/ROCK pathway phosphorylates MYPT1, resulting in a decrease in MLCP activity. High [K^+^] stimulation increased MYPT1 phosphorylation at the two ROCK sites, Thr697 and Thr855, after stimulation for 15 min. In other words, high [K^+^]‐induced MYPT1 phosphorylation at Thr697 and Thr855 increased only during the tonic component of high [K^+^]‐induced contraction. These results are consistent with the time course of RhoA activation by high [K^+^] stimulation, which we reported previously (Mills, Mita, Nakagawa, et al., 2015; Mita et al., 2013). Additionally, these increases in MYPT1 phosphorylation were completely inhibited by Rhosin but not Y16 (Figure 4). These results suggest that the inhibition of Pyk2‐mediated MLCP activity induced by high [K^+^] stimulation is caused by MYPT1 phosphorylation at Thr697 and Thr855 via Y16‐insensitive RhoGEF‐mediated activation of RhoA/ROCK, leading to an increase in LC_20_ phosphorylation and the tonic component of contraction. However, pre‐treatment of arterial strips with Rhosin also reduced the phasic component of the contraction induced by 60 mM K^+^ (Figure 1). This means that the inhibitory effect of Rhosin on the phasic component of the contraction may involve a mechanism different from the activation of the RhoA/ROCK pathway through Pyk2.

Angiotensin (Ang) II‐induced LARG‐RhoA complex formation in cultured vascular smooth muscle cells of spontaneously hypertensive rats (SHR) was significantly inhibited at 50 μM Y16 and phosphorylation of MYPT1 at 5 μM or more (Chiu et al., 2021). However, in this study, Y16 had little effect on 60 mM K^+^‐induced contraction (Figure. 2) and MYPT1 phosphorylation at Thr697 and Thr855 (Figure. 4). Regulation of vascular smooth muscle contraction involves multiple mediators, many of which act through G protein‐coupled receptors (GPCRs) on vascular smooth muscle cells (Maguire & Davenport, 2005). Receptors that mediate vasoconstriction couple with the G proteins Gq/G_11_ and G_12_/G_13_ to stimulate phosphorylation of LC_20_ via MLCK‐ and RhoA/ROCK‐mediated signaling pathways, respectively (Gohla et al., 2000; Maguire & Davenport, 2005; Somlyo & Somlyo, 2003). It is known that activation of the RhoA/ROCK pathway by the stimulation of GPCRs coupling to Gα_12/13_ leads to phosphorylation of LC_20_ through inhibition of MLCP (Strassheim et al., 2019). Moreover, it was reported that LARG, p115‐RhoGEF, and PDZ‐RhoGEF, which act upstream of the RhoA/ROCK pathway, are regulated by Gα_12/13_ following Angiotensin II AT_1_ receptor activation (Chikumi et al., 2004; Chiu et al., 2012; Shang et al., 2013). Therefore, the discrepancy between our present results and those by Chiu et al. (Chiu et al., 2021) may be due to differences in the mechanisms of RhoGEFs activation by GPCR stimulation and membrane depolarization without receptor stimulation. Furthermore, when examining the synergistic effect between Rhosin and Y16, the contraction induced by 60 mM K^+^ was not affected, compared to the inhibitory effect in the presence of Rhosin alone (Figure 3). These results indicated that there is not a synergistic effect between Rhosin and Y16 on K^+^‐induced contraction and Y16 cannot cooperate with Rhosin to inhibit Y16‐sensitive RhoGEFs‐RhoA interaction and RhoA activation in high [K^+^]‐induced Ca^2+^ sensitization. Y16 has been reported to inhibit RhoA interaction with other RGS domain‐containing RhoGEFs such as LARG and p115‐RhoGEF (Shang et al., 2013). Therefore, it seems that Y16‐sensitive RhoGEFs, such as LARG and PDZ‐RhoGEF, may not be involved in the Pyk2‐mediated Ca^2+^‐dependent RhoA/ROCK pathway in membrane depolarization‐induced‐sustained contraction of rat caudal arterial smooth muscle.

Pyk2 is a non‐receptor tyrosine kinase regulated by various extracellular signals and is activated by increasing [Ca^2+^]_i_ (Lipinski & Loftus, 2010). We previously reported that depolarization‐induced Ca^2+^ influx activates Pyk2 upstream of the RhoA/ROCK pathway, leading to phosphorylation of MYPT1 and inhibition of MLCP, which results in sustained elevation of LC_20_ phosphorylation that is responsible for the sustained contraction in response to membrane depolarization (Mills, Mita, Nakagawa, et al., 2015; Mita et al., 2013). However, the mechanism by which Pyk2 activates RhoA remains to be elucidated. Transfection studies using cultured smooth muscle cells indicated that PDZ‐RhoGEF could link activated Pyk2 to RhoA activation via RhoGEF phosphorylation and activation (Ying et al., 2009). Furthermore, it was reported that the association of Pyk2 with tyrosine‐phosphorylated PDZ‐RhoGEF is enhanced on Pyk2 activation by Ang II, suggesting that Ang II‐activated Pyk2 induces the tyrosine phosphorylation of PDZ‐RhoGEF and activates the RhoA/ROCK pathway in smooth muscle cells (Ohtsu et al., 2005). It was also reported that the activation of PDZ‐RhoGEF and LARG is essential for the sustained vascular smooth muscle contraction caused by the thromboxane A_2_ receptor agonist U46619 and endothelin‐1 (Artamonov et al., 2013). We have confirmed that several RhoGEFs, including LARG and PDZ‐RhoGEF, and several RhoGAPs are expressed at both mRNA and protein levels in rat caudal arterial smooth muscle (KA). However, we did not yet identify specific RhoGEFs coupled with Pyk2 in this study. These results suggest that PDZ‐RhoGEF and/or LARG is less involved in RhoA/ROCK activation via membrane depolarization‐induced Pyk2 activation in caudal arterial smooth muscle.

**FIGURE 5:**
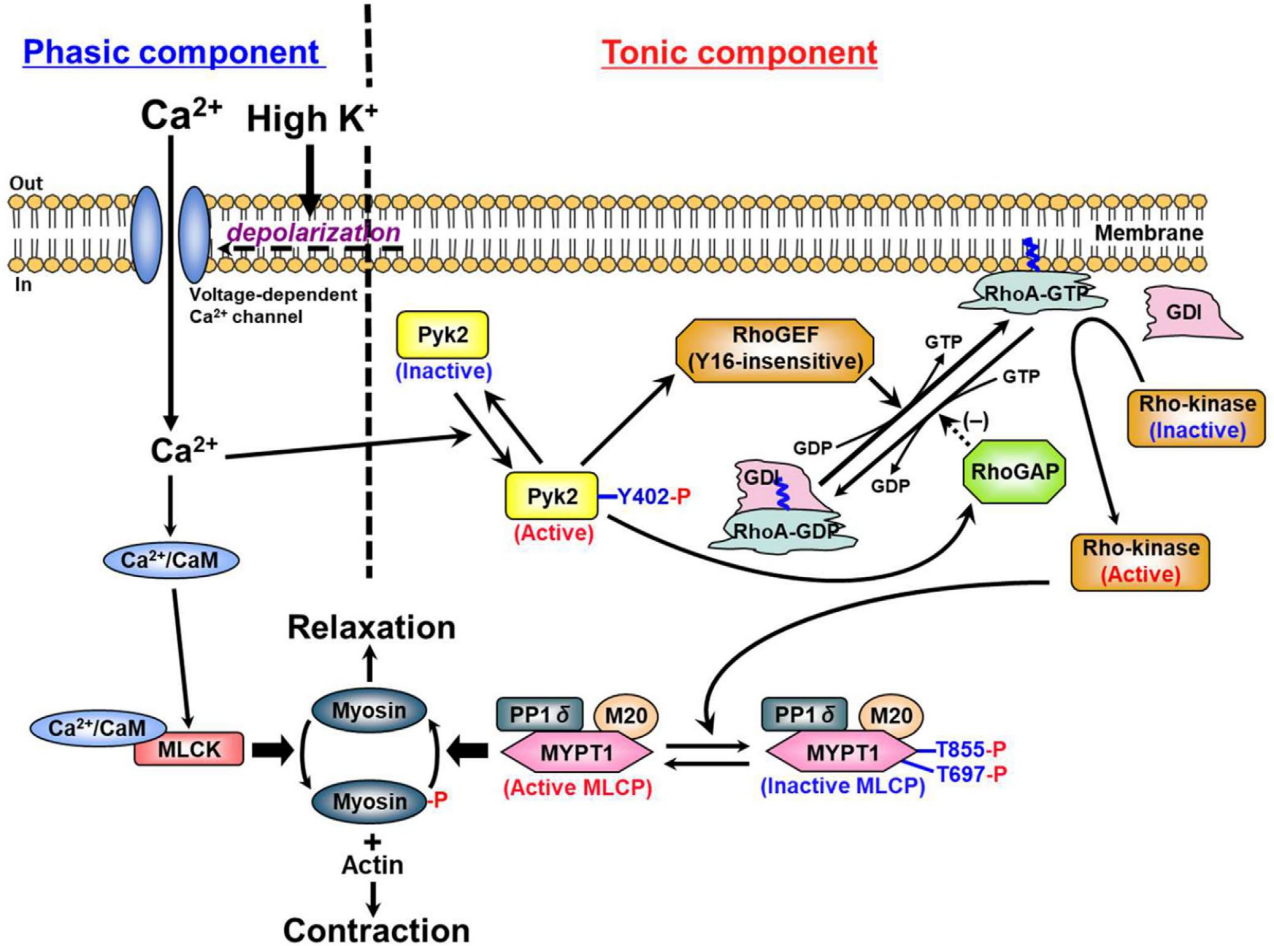
The mechanism of membrane depolarization‐induced contraction of vascular smooth muscle contraction elicited by membrane depolarization comprises two phases: (i) the phasic component involves phosphorylation of myosin by Ca^2+^/CaM‐dependent MLCK and (ii) the tonic component involves phosphorylation of MYPT1 by activation of the RhoA/ROCK pathway via activation of Y16‐insensitive RhoGEFs and/or inhibition of RhoGAPs by Pyk2. CaM, calmodulin; GDI, GDP dissociation inhibitor; GDP, guanosine diphosphate; GTP, guanosine triphosphate; M20, a 20 kDa regulatory subunit of MLCP; MLCK, myosin light chain kinase; MLCP, myosin light chain phosphatase; MYPT1, myosin‐targeting subunit of MLCP; PP1cδ, a 38 kDa catalytic subunit of type 1 protein phosphatase δ isoform; Pyk2, proline‐rich tyrosine kinase 2; RhoGAP, Rho GTPase‐activating protein; RhoGEF, Rho guanine nucleotide exchange factor.

The following principal conclusions are reached from this study (Figure 5): (i) The tonic component of membrane depolarization‐induced contraction without receptor stimulation involves the mechanism of RhoA/ROCK activation through Ca^2+^‐dependent activation of Pyk2 by Ca^2+^ influx. (ii) Activated Pyk2 activates RhoGEFs and/or inhibits RhoGAPs, resulting in activation of the RhoA/ROCK pathway. (iii) Pyk2‐activated RhoGEFs cannot catalyze RhoA when inhibited by Rhosin and are Y16‐insensitive RhoGEFs. (iv) This mechanism of RhoGEFs‐mediated RhoA/ROCK activation is distinct from that mediated by receptor stimulation. (v) The RhoA/ROCK pathway activated by Pyk2 phosphorylates MYPT1 and suppresses its activity, thereby causing sustained contraction induced by membrane depolarization. Overactivation of the RhoA/ROCK pathway has been reported to be involved in the etiology of hypertension (Uehata et al., 1997). Consequently, Pyk2 may be a new therapeutic target for cardiovascular disease treatment by controlling vascular smooth muscle hypercontractility. We are now investigating the identification of RhoGEFs or RhoGAPs that couple with Pyk2 in caudal arterial smooth muscle.

## FUNDING INFORMATION

This work was supported by JSPS KAKENHI Grant Number JP19K07108 to MM.

## CONFLICT OF INTEREST STATEMENT

The authors declare no conflict of interest.

## Data Availability

All the raw data are available from the corresponding author upon reasonable request.
